# Clue Cells and Pseudo Clue Cells in Different Morphotypes of Bacterial Vaginosis

**DOI:** 10.3389/fcimb.2022.905739

**Published:** 2022-05-27

**Authors:** Alexander Swidsinski, Vera Loening-Baucke, Sonja Swidsinski, Jack D. Sobel, Yvonne Dörffel, Alexander Guschin

**Affiliations:** ^1^ Medizinische Klinik, Charité Charite Campus Mitte (CCM), Universitätsmedizin, Berlin, Germany; ^2^ Institute of Molecular Medicine, Sechenov First Moscow State Medical University, Moscow, Russia; ^3^ Microbiology, MDI Limbach GmbH, Berlin, Germany; ^4^ Wayne State University School of Medicine, Detroit, MI, United States; ^5^ Outpatient Clinic, Charité Universitätsmedizin Berlin, Charite Campus Mitte (CCM), Berlin, Germany; ^6^ Moscow Scientific and Practical Center of Dermatovenerology and Cosmetology, Moscow, Russia

**Keywords:** dysbiosis, FISH, clue cells, bacterial vaginosis, biofilm vaginosis, bacterial excess vaginosis, polymicrobials

## Abstract

**Introduction:**

Clue cells (epithelial cells heavily covered with adherent bacteria) are an accepted clue to the diagnosis of bacterial vaginosis. However, the exact morphologic criteria of clue cells and bacterial adherence were never elaborated.

**Materials and Methods:**

We investigated adhesive and cohesive patterns of main microbiota groups in vaginal discharge using fluorescence *in situ* hybridization (FISH). Samples from 500 women diagnosed with bacterial vaginosis and positive for clue cells with classic microscopy were collected from 42 gynecologic practices in Berlin and reexamined in our FISH laboratory for the spatial distribution of Bifidobacteriaceae, *Gardnerella*, ***Fannyhessea vaginae*** (*Atopobium*); low G+C (guanine+cytosine) bacteria, lactobacilli, *Lactobacillus iners*; *Lactobacillus crispatus*, Gamma-Proteobacteria; and Enterobacteriaceae, *Prevotella–Bacteroides*, *Veillonella*, and *Coriobacterium* groups.

**Results:**

Bacterial taxa present in vaginal smears were not accidentally assembled according to their relative abundance but were built in group-specific distribution patterns, which can be well described by two features: cohesiveness to each other and adherence to epithelial cells. Accordingly, four patterns can be distinguished: dispersed (non-adherent bacteria), dispersed adherent bacteria, cohesive (non-adherent) bacteria, and cohesive adherent bacteria. Direct cohesive adherence to the epithelial cells representing true clue cells was unique for *Gardnerella* species and observed only in 56% of the investigated samples. In the remaining vaginal samples, the epithelial cells were mechanically entrapped in bacterial masses, and the composition was unrelated to the epithelial cell surface, building non-adherent pseudo clue cells. The proportion of women with true clue cells in their samples from different gynecologic practices varied from 19% to 80%.

**Discussion:**

Taxon indifferent imaging is inadequate for the exact analysis of the microbial layer adjacent to the vaginal epithelial cells. Morphologically seen bacterial vaginosis is a mix of at least two different conditions: biofilm vaginosis and bacterial excess vaginosis.

## Introduction

Excessive vaginal discharge, which is troublesome, is the most frequent complaint in sexually active women visiting gynecologists (Redelinghuys et al., 2020). Only a small portion of such cases are attributed to sexually transmittable or other exactly defined mono-infections (Peebles et al., 2019). In most cases of bacterial vaginosis (BV), a marked increase in bacterial diversity and concentrations is observed without apparent cause. The composition of microbiota in such overgrowth dysbiosis is highly variable in patients, and even samples were taken from the same patient at different time points (Peebles et al., 2019; Muzny et al., 2019; Redelinghuys et al., 2020).

Gardner and Dukes in 1955 described epithelial cells covered with a bacterial layer in vaginal discharge of symptomatic women, which were absent in healthy women, and called them clue cells for their central role in recognizing the condition (Gardner and Dukes, 1955). Since then, many other researchers confirmed the vital role of clue cells in the diagnosis of the dysbiotic condition that was later named bacterial vaginosis. Despite broad acceptance and more than 60 years passing since the first description, no consensus exists about what should be regarded as a clue cell (Muzny et al., 2019; Redelinghuys et al., 2020). Exact criteria defining clue cells were never elaborated. Each investigator has his own highly subjective and even individually not reproducible feeling of what a clue cell already is and what it is still not. The main difficulty is the high heterogeneity of the microbial cover in wet smear or Gram/Pap stain microscopy. Transient findings between an obvious clue cell and a definitively normal epithelial cell are much more numerous than the both “unmistakable” extremes and still impossible to assign according to classic definition. As a solution, it was proposed to accept as clue cells only findings in which 20% of all visualized epithelial cells were covered with bacteria. But such a solution is not better and has the same deficiency as the detection of a “significant number” of clue cells.

The aim of the present study was to investigate how different bacterial groups contribute to the clue cell adhesion using fluorescence *in situ* hybridization (FISH).

## Patients, Materials, and Methods

The study was based on vaginal samples from 500 (19- to 51-year-old women with a mean age of 28 years) symptomatic white women. Probes were randomly selected from 42 Berlin gynecologic practices with previous observation of positive clue cells with classic microscopy, Amsel criteria confirmed BV, and exclusion of sexually transmitted diseases or candidiasis. No other exclusion criteria including the previous therapy were applied since the most important fact for us was the presence of clue cells, diagnosed by other physicians. Therefore, usual exclusion criteria like previous therapy, and racial and social heterogeneities were not important or applied.

Nine gynecologic practices delivered more than 20 samples each, resulting in 271 samples. The number of samples from the remainder offices varied between 3 and 17 (mean 7).

ESwab™ 493C02 (COPAN Diagnostics, Murrieta, CA, USA) was used for the collection of vaginal smears and stored at 4°C between 12 and 48 h prior to FISH, whereby 43/500 samples were forwarded to FISH analysis within 12 h and 122/500 within 24 h after sampling. The samples were fixated with Carnoy’s solution according to the protocol (Swidsinski and Loening-Baucke, 2017).

Amies smears were gently vortexed. The suspension measuring 100 µl was fixated by adding 1,000 µl of Carnoy’s solution (alcohol/chloroform/acetic acid 6/3/1 by volume) (Meier et al., 1999) for 10 min. The samples were then centrifuged at 6,000 RPM for 8 min and stored in 50 µl of Carnoy’s solution.

### Fluorescence *In Situ* Hybridization

Fields of 10 mm × 10 mm were marked on SuperFrost slides (Langenbrinck, Emmendingen, Germany) with a PAP pen (Kisker-Biotech, Steinfurt, Germany). Aliquots of vortexed fixed vaginal swab suspension measuring 5 μl were dropped onto the marked field. The slides were dried for 60 min at 50°C before FISH analysis.

The exact protocols with single steps and solutions are also presented at http://www.swidsinski.de/zusatzdateien/fishmethode/fishmethode.htm.

The following probes were hybridized with all vaginal samples:

Bif 164 (Bifidobacteriaceae), Gard662 (*Gardnerella*), Ato291 (*Atopobium*) (Swidsinski et al., 2019),

LGC35 (low guanine+cytosine bacteria including Mycoplasmatales, Firmicutes, Bacillales, Lactobacillales) (Meier et al., 1999), Lab158 (lactobacilli) Liner23-2 (*Lactobacillus iners*), Lcrips16-1 (*Lactobacillus crispatus*) (Swidsinski et al., 2019), GAM42a (Gammaproteobacteria) (Amann et al., 1990), Ebac1790 (Enterobacteriaceae) (Bohnert et al., 2000), Bact1080 (*Prevotella–Bacteroides*) (Doré et al., 1998), Veil223 (*Veillonella*) (Harmsen et al., 2002), and Cor653 (Coriobacteriaceae) (Harmsen et al., 2000).

For multicolor analysis, each of the applied oligonucleotide probes was synthesized with a carbocyanine Cy3 (orange) and Cy5 (dark red) fluorescent dye. The hybridizations were performed at 50°C as previously described (Swidsinski and Loening-Baucke, 2017). DAPI stain was used to visualize the DNA-rich structures of bacteria and eukaryotic cells.

The Cy3-stained probe served as the evaluation of the targeted bacterial cluster, Cy5-stained probe served as a reference to the taxonomically broader microbiome group.

Despite previously tested and reported specificity of the FISH probes, we do know from our own experience that the reported specificity is never absolute since the specificity testing usually involves only isolated cultured strains. However, the real diversity of naturally occurring microorganisms is much higher than that of the cultured representatives. Therefore, it was important to support the hybridization results through a comparison of hybridization data of related and taxonomically unrelated FISH probes in multicolor FISH. Only hybridizations in which bacteria hybridized with Bif universal and *Gardnerella* probes, but did not hybridize with FISH probes representing other specificities, were regarded as genuine. Within the sets of our experiment, we did not observe any cross hybridizations of bacteria with unrelated microbial probes.

A Nikon e600 fluorescence microscope was used. The images were photo-documented with a Nikon DXM 1200F color camera and software (Nikon, Tokyo, Japan).

## Results

### Patterns of Bacterial Distribution in Vaginal Smears

In the majority of the samples, the microbial groups within the vaginal smear were not accidentally mixed and evenly distributed over glass slides; on the contrary, each taxon demonstrated its own difference from other spatial profiles and relation to the epithelial cells. According to these, bacteria could be divided into the following: diffusely distributed with clear distances between each other, only seldom building clumps of about 30 bacteria or less; bacteria cohesively attached to each other without spaces between them and preferentially organized in groups of 30 or more bacteria (up to 1,000–2,000) each. The transition from dispersed to cohesive condition was not abrupt. In each case, a considerable part of the cohesive bacteria was also distributed in the surroundings and vice versa. As cutoff, we regarded bacteria as cohesive only when at least 10 clumps with 30 bacteria each or one or more clumps together exceeded 300 coherent bacteria and were detected in the whole microscopic surface available for evaluation. Bacteria could be divided into the desquamated epithelial cells in non-adherent (having similar or higher concentrations in the distance than on the epithelial cell surface) and adherent bacteria (attached to epithelial cells in concentrations exceeding at least twice the concentrations of the same bacteria in the surroundings). In the case of very-high-density cohesive bacteria (with no spaces between microorganisms), exact concentrations could not be quantified. In such cases, a surface completely occupied by bacteria in the area of 10 µm adjacent to the epithelial surface was compared to regions at a distance of more than 20 µm from the epithelial surface. The total surface of the cohesive microbial layer attached to the epithelial cell surface and adjacent areas had to exceed the surface not connected to the epithelial cells clumps at least by a factor of two.

The adherence does not involve all epithelial cells. To make data numerically comparable, we arbitrarily defined bacteria as adherent when at least 10 epithelial cells within a smear demonstrated clear adherence as described above.

The investigations were performed at resolutions between ×100 and ×1,000. The combination of cohesiveness and adherence revealed four patterns of spatial distribution: dispersed (non-adherent), dispersed-adherent, cohesive (non-adherent) sludge-like, and cohesive-adherent or biofilm-like.

For convenience, we do not mention “non-adherent” in the following presentation, when not necessary for understanding.

**Figure 1:**
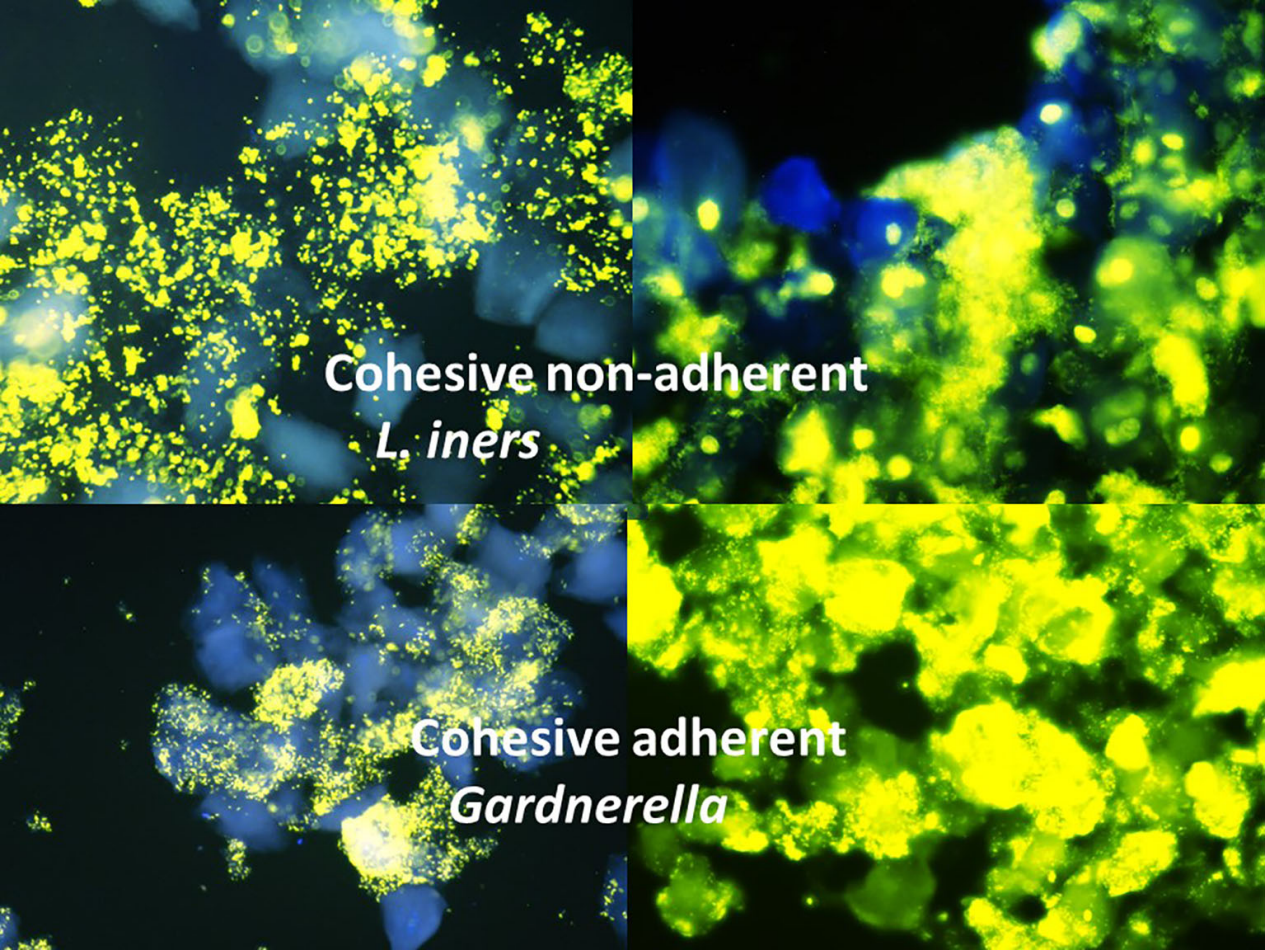
Four examples of cohesive adherent (*Gardnerella with moderate and high density*) and cohesive non-adherent (*Lactobacillus iners* with moderate and high density) spatial distribution of bacteria; C3 yellow fluorescence against blue DAPI counterstain background, ×400.

Cohesive-adhesive growth was typical for biofilms attached to the epithelial cell surface and built true “clue cells” (**Figure 1**). The microbial cover of such cells was identically assembled all over the microscopy surface but varied in intensity from cell to cell, leaving some epithelial cells completely free. However, a finding of at least 10 epithelial cells with identical structured cohesive adherent cover was sufficient and unmistakable for the identification of a cohesive-adherent pattern, even in cases where the overall number of bacteria and epithelial cells on the glass slide was moderate (**Figure 1**, top-left image).

In contrast, dispersed and cohesive non-adherent growing microorganisms embedded epithelial cells only secondarily (**Figure 1**, bottom images). Even when highly concentrated and completely enclosing multiple or even most of the available epithelial cells through surrounding microbial growth, the resulting microbial cover of single epithelial cells remained unique for each epithelial cell and was not reproducible.

**Graph 1:**
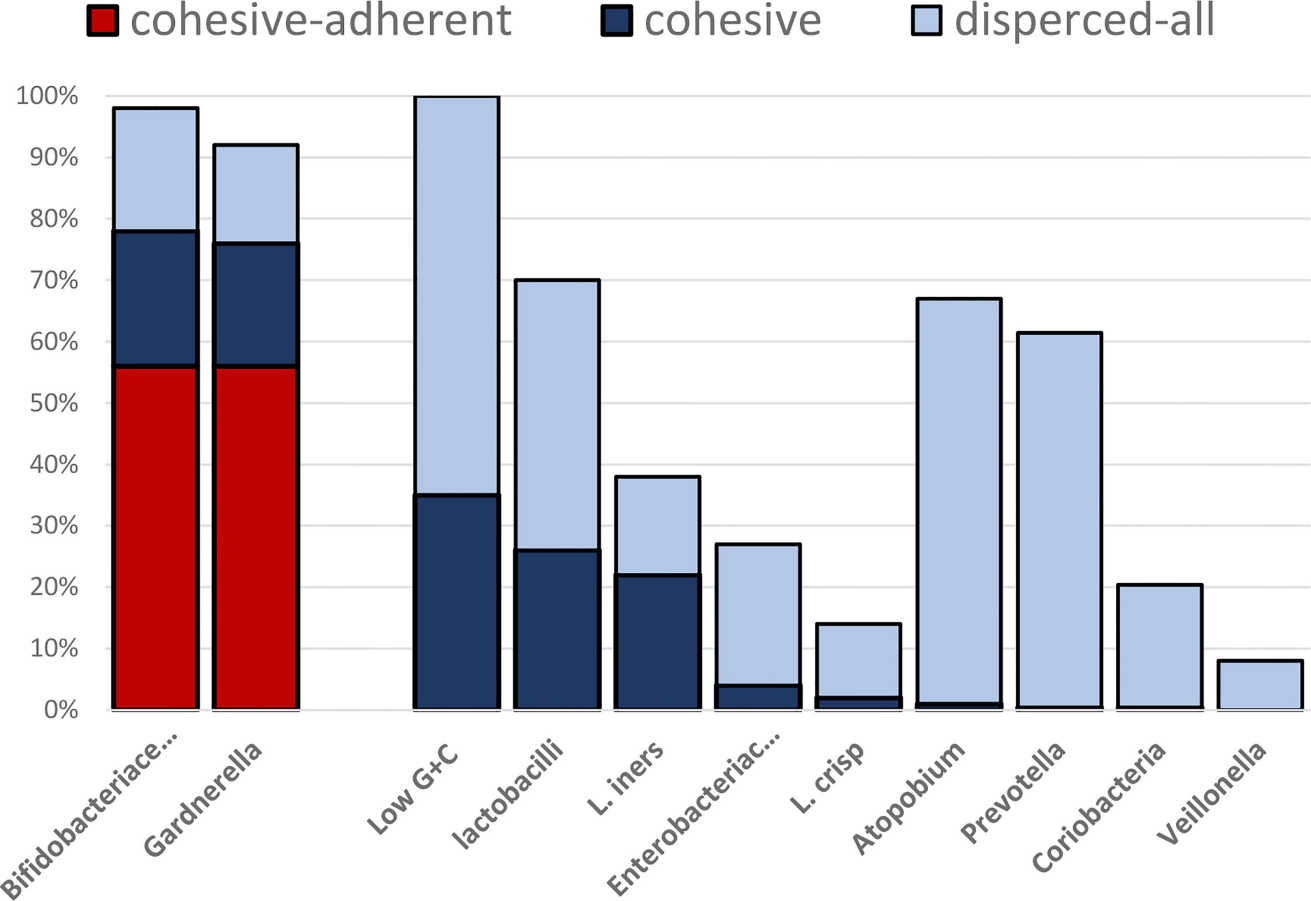
Percent of samples demonstrating cohesive adherent, cohesive non-adherent, and dispersed patterns of spatial distribution for each of the investigated microbial groups. The exact numbers are presented in **Table 1** (as additional material only).

**Graph 1** and **Table 1** demonstrate taxon-specific patterns of spatial microbial distribution.

**Table 1:** Number of patients/samples demonstrating different patterns of spatial distribution for each of the investigated groups.

	Dispersed	Dispersed adherent	Cohesive	Cohesive adherent	All
					500
Bif164	47	52	109	281	489
Gard662	34	45	98	278	455
LGC35	223	101	176		500
Lab158	120	100	131		351
Liners23-2	41	39	108		188
Lcrisp16-1	34	25	12		71
^*^Gam42a	84	31	21		136
Ebac1790	84	31	21		136
Ato291	205	124	4		333
Bacto1080	269	37	2		308
Cor653	102	2	2		106
Veil223	42	2			44

^*^Gam42a and Ebac1790 probes hybridized with the same bacteria indicating that except for Enterobacteriaceae, no other Gammaproteobacteria are present in vaginal samples.

Dispersed distribution was the most common and could be observed for all main representatives of the vaginal microbiota (**Table 2**). ***Fannyhessea vaginae*** (*Atopobium*), Enterobacteriaceae, *Prevotella*, *Veillonella*, and *Coriobacterium* groups were nearly exclusively dispersed. Cohesiveness was typical for *Gardnerella* and progressively declined from low GC bacteria to lactobacilli *L. iners* and *L. crispatus*, with Enterobacteriaceae ***F. vaginae*** and *Prevotella* being only exceptionally cohesive. Although low guanine+cytosine bacteria/lactobacilli and specifically *L. iners* could grow to extended cohesive fields covering large surfaces and include multiple epithelial cells in this growth, no cohesive adherence was observed in these groups. The highest concentrations of bacteria were always located outside of the epithelial cell or regions adjacent to them, building secondary pseudo clue cells; such a bacterial cover was definitively non-adherent.

**Table 2:** Morphological forms of bacterial vaginosis (BV) based on this study.

	Mode of growth	FISH appearance	Taxa
Biofilm vaginosis (BiV)	Epithelium attached biofilm growth	Cohesive biofilm adherent to and presumably growing on epithelial cells or true clue cells	*Gardnerella* spp.
Bacterial excess vaginosis (BeV)	Sludge like growth within vaginal slime	Cohesive and diffuse bacteria mainly growing on its own and only secondarily incorporating epithelial cells into the bacterial masses building pseudo clue cells	*Lactobacillus iners* and other lactobacilli Enterobacteriaceae
BV modifications	Diffuse or in isolated islands mixed with other bacteria	Non-coherent growing microorganism using overgrowth of other bacteria for own propagation	*Atopobium* (*Fannyhessea vaginae*), probably *Mobiluncus*, *Mycoplasma* spp., *Candida*, etc., not investigated in this study

FISH, fluorescence in situ hybridization.

A cohesive-adhesive pattern of distribution was unique for Bifidobacteriaceae (exclusively represented by *Gardnerella*) and comprised 56% of all investigated vaginal samples. The fluorescence of the Bif164 probe was however higher and visually more perceptible.

The difference between Bifidobacteriaceae and *Gardnerella* was altogether small and increased from coherent non-adherent to disperse-adherent and dispersed growing *Gardnerella*, indicating that in these cases other than *Gardnerella*, Bifidobacteriaceae species or other *Gardnerella* genotypes could be involved (**Table 2**).

The discrimination between cohesive adherent *Gardnerella* biofilms primarily enwrapping true clue cells and all other spatial patterns of microbial distribution was straightforward and unmistakable (**Figure 1**). These were especially obvious in cases with mainly dispersed growing bacteria such as ***F. vaginae*** (*Atopobium*). Although ***F. vaginae*** bacteria were often concomitant to cohesive *Gardnerella* and preferentially located within clue cell cover, different from *Gardnerella*, they always grew dispersedly or distributed in small islands within more abandoned *Gardnerella* conglomerates and never adhered directly to the epithelial cell surface (not shown).

**Graph 2:**
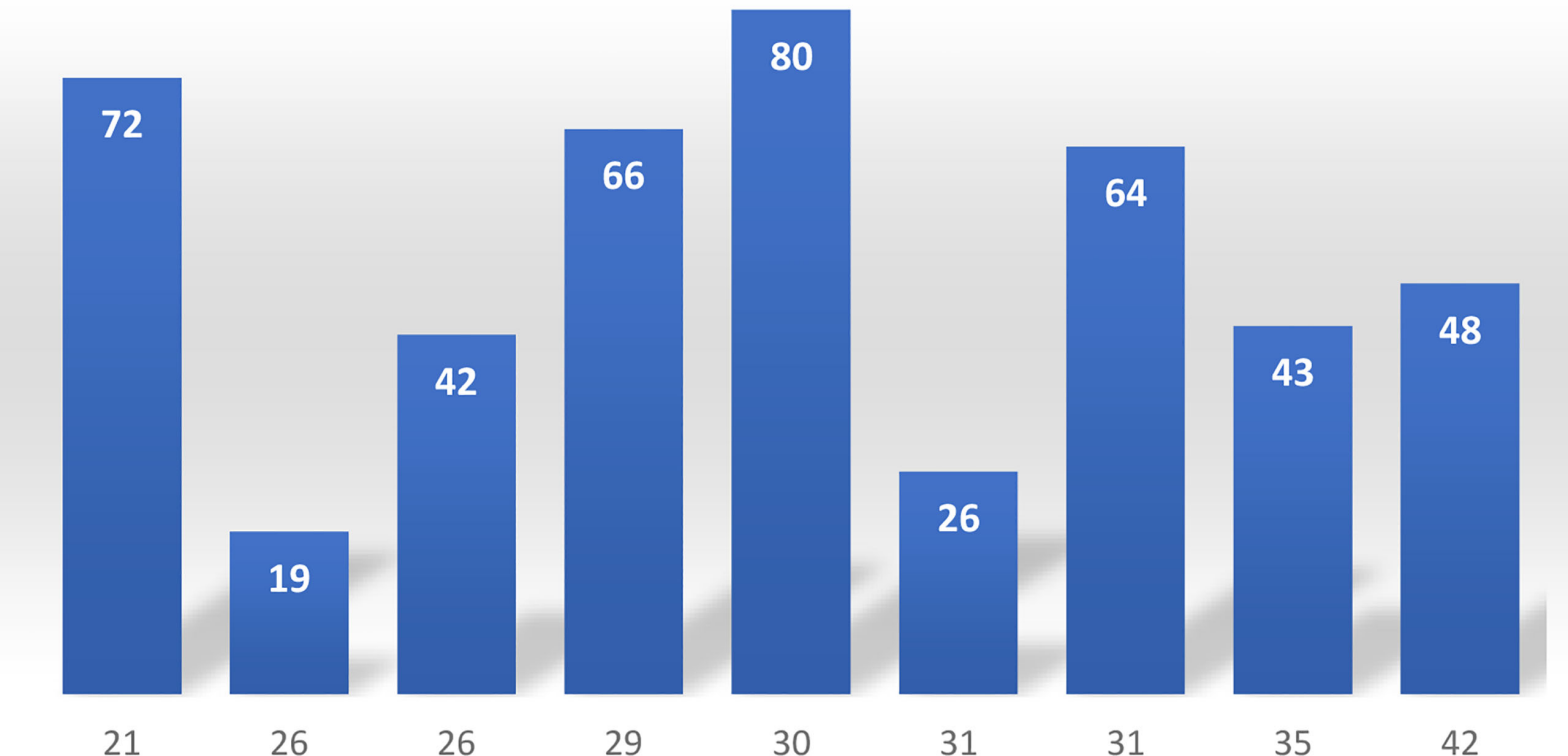
Percent of patients (vertical column) with cohesive-adhesive clue cells in samples from 9 gynecologic practices, delivering more than 20 samples each (N = horizontal line).

On the other hand, the agreement between FISH data and the taxon unrelated methods was low, and we found true clue cells only in 56% of the vaginal samples, which were previously identified as positive for clue cells with classic microscopy. Moreover, the proportion of cohesive adherent “clue cells” in samples sent from different gynecologic practices varied highly, ranging between 19% and 80% (**Graph 2**).

## Discussion

Our data demonstrate that unfortunately there is no “cheap reliable and convenient” way to recognize clue cells by classic microscopy. The definition of “stippled bacterial cover obscuring epithelial cell margins” is highly subjective. The uniform appearance of bacterial aggregates is erroneous. Taxonomic decoding of microorganisms by FISH unravels the highly differentiated structure of the bacterial cover and indicates the existence of at least two principally different modes of its formation: characteristic adherent growth of cohesive *Gardnerella* species on the surface of the epithelial cells leading to the development of true “clue cells” and sludge-like growth of individually arranged bacterial groups in the vaginal slime, which appeared to be “pseudo clue cells.” They appeared to be secondarily entrapped (enveloped) and were in the way of epithelial cells in regions of microbial excess.

The characteristic adherent growth of cohesive *Gardnerella* species on the surface of the epithelial cells represented biofilm growth and should be probably more accurately called biofilm vaginosis (BiV). A proper name for the second condition could be a bacterial excess vaginosis (BeV). For a better overview, we present the proposed definitions in **Table 1** separately. The extension of the name by “excess” might be reasonable since it allows a separation of high microbial load conditions from aerobic and desquamative vaginal dysbiosis with overall low microbial counts independent of the presence or absence of pseudo clue cells.

The BiV was highly consistent in its morphologic appearance, while bacterial overgrowth vaginosis was mainly a mismatch of diversely distributed microbial groups. However, within the latter, some bacterial groups may dominate the picture with Enterobacteriaceae covering the microscopic surface in 4% of samples, building an eye-catching blanket or with *L. iners* arranged in 22% of samples in extensive cohesive conglomerates with a characteristic appearance. Obviously, these microorganisms did not need a firm surface for proliferation and preferred to grow freely suspended in slime. A situation could be basic and rooted in the natural history of polymicrobial communities of which some were specialized to grow attached to firm surfaces and form biofilms like stromatolites or vaginal biofilms (Swidsinski et al., 2014) and others were swimming free in fluids forming activated sludge (Mesquita et al., 2013). It is therefore and also imaginable that BeV could be further subdivided into etiologically relevant subgroups according to organisms leading the sludge-like microbial proliferation, of which cohesive *L. iners* could be of special interest not only for its high frequency in vaginal dysbiosis but also for the increasingly reported clinical significance (Zheng et al., 2021). A possible name for such could be *L. iners* excess vaginosis.

We found no other taxon-characteristic appearance features in our series, but the number of 500 samples may be too small to uncover a variety of dysbiotic morphology.

However, hybridization with the LGC35 probe demonstrated the highest occurrence of cohesive non-adherent bacteria that formed large sludge aggregates. These findings are less relevant than the hybridization with the *L. iners* probe. The low guanine+cytosine group is (similarly to Gram stain) very broad and includes Mycoplasmatales, Firmicutes, Bacillales, and Lactobacillales (Meier et al., 1999). The cohesive non-adherent bacteria detected by the LGC probe might be in reality a composite mishmash of non-prevalent bacterial groups being truly cohesive only in the case of *Lactobacillus* species. However, the absence of adhesive coherent growth in all vaginal samples when investigated with LGC35-FISH stresses the importance of *Gardnerella* species as the only microorganisms important for biofilm formation.

In contrast to the LGC35 probe, the use of the broad Gam42a probe that covered the phylum of Gammaproteobacteria added nothing to the hybridizations with the Enterobacteriaceae-specific probe, indicating that besides Enterobacteriaceae, no other Gammaproteobacteria were involved in shaping vaginal dysbiosis conglomerates.

Whichever terms and classifications ultimately prevail in the future, one thing is for certain: the syndrome currently diagnosed as BV is actually highly heterogeneous and made up of morphologically different conditions. All of the samples included in our study and obtained in different gynecologic practices contained clue cells routinely diagnosed with classic microscopy. Although we confirmed true clue cells in 56% of the samples, this value cannot be generalized for smaller groups and groups selected for specific criteria. Unfortunately, the comparison of nine practices that delivered more than 20 samples revealed an occurrence rate of true clue cells that ranged between 19% and 80%.

This heterogeneity could be an explanation for the previously observed inconsistencies in results of clinical studies regarding sexual transmissibility, severity, and incidence of associated complications, and also inconclusive therapeutic outcomes in women with BV (Muzny et al., 2019; Coudray and Madhivanan, 2020). The composition in contrasting vaginosis subtypes would predictably differ depending on the clinical focus, selection criteria, country, and even region of the performed study. Future clinical investigations should differentiate the cohesive-adhesive microbiome subtypes in their groups to ensure that they are not comparing apples and oranges.

### Concluding Comments and Limitations

Unfortunately, FISH is not available in all laboratories, which limits the broad application of such a diagnostic method. FISH would probably remain indispensable for such differentiation, as long as the exact biomarkers responsible for cohesive adherent biofilm growth of *Gardnerella* are unknown.

However, for routine use, a one-step multicolor hybridization with Bif164-C3 probe together with LGC-C5 probe should be sufficient to distinguish the main morphotypes.

The use of the universal for Bifidobacteriaceae Bif164 probe for such diagnostic is preferable for the following reasons: Bifidobacteriaceae other than *Gardnerella* are numerically marginal in the vaginal microbiome. The accidental presence of *Bifidobacteria* other than *Gardnerella* in the vagina cannot therefore bias the final results. On the other hand, the genetic and taxonomic heterogeneities of *Gardnerella* species involved in BV are enormous. Each year, new yet unknown sequences of isolated species are reported. It would be important to include them all with routine testing. The Bif164 probe demonstrates a high luminescence and does not cross hybridize with other vaginal bacteria that positively hybridize with other probes used in the series including the ***F. vaginae*** (*Atopobium*) probe. Although the Bif164 probe often hybridizes with dispersed *Gardnerella*, the same is true for the *Gardnerella* probe. It demonstrates that genotypes and/or specific genes responsible for cohesive adherent growth are still to be found.

We also consider the dark red C5-stained LGC35 probe as a valuable addition for routine FISH diagnostics. The LGC35 probe covers all lactobacilli and also many other non-*Gardnerella* vaginal microbiota and is therefore preferable for the detection of cohesive non-adherent bacterial overgrowth. For specific questions, the LGC probe can be substituted by Liners23-2 or by an extended set of other available FISH probes.

The costs of consumables for one-shot FISH analysis are the same as for classic Gram stain; however, for manual performance, 30 additional minutes must be taken into account. The one-time costs for procurement of microscope and camera of about 40,000 € are likewise necessary (present in each large laboratory). Investigators experienced in saline wet mount or Gram stain microscopy would have no difficulties in using FISH.

Some of the results of our study could be partially due to the long storage time of some samples, exceeding 24 h; the proportion of true clue cells in 102 samples delivered on the same day was 54% and was the same as in all other samples. However, this limitation had to be taken into account to access the cross section of all gynecologic practices in Berlin.

One important restraint of all FISH studies is the still limited number of available in open-access FISH probes. For some of the important species such as *Mycoplasma*, no satisfactory working FISH probes have been developed thus far. One can hope that such limitations are temporary and that the range offered for species-specific FISH probes will increase.

Finally, the clinical significance of our morphologic FISH microscopy-based subtypes of BV is unknown, but our findings emphasize the heterogeneity of this common syndrome.

## Data Availability Statement

The raw data supporting the conclusions of this article will be made available by the authors, without undue reservation.

## Ethics Statement

The studies involving human participants were reviewed and approved by the ethical committee of the Charité approval number EA1/088110. The patients/participants provided their written informed consent to participate in this study.

## Author Contributions

Each author named in the byline participated actively and sufficiently in the study. AS, AG, and YD designed the study. AS and SS conducted the study. JS and VL-B critically revised the manuscript. AS and YD performed the FISH. AS and SS analyzed the data. All authors contributed to the conception of the work, revising the data, shaping the manuscript, and approving the final draft submitted.

## Funding

The study was supported by the Charité University research promotion grant (2016) and the German Federation of Industrial Research Associations ZIM Project ZF4143701AJ5.

## Disclaimer

Both funding sources were not involved in the study design, collection, analysis, and interpretation of data, in writing a report, or the decision to submit the article for publication.

## Conflict of Interest

Author SS was employed by MDI Limbach GmbH.

The remaining authors declare that the research was conducted in the absence of any commercial or financial relationships that could be construed as a potential conflict of interest.

## Publisher’s Note

All claims expressed in this article are solely those of the authors and do not necessarily represent those of their affiliated organizations, or those of the publisher, the editors and the reviewers. Any product that may be evaluated in this article, or claim that may be made by its manufacturer, is not guaranteed or endorsed by the publisher.
